# Coexisting ADAR and TSHB Mutations in an Infant With Retinal Detachment and Transient Cardiomyopathy

**DOI:** 10.1155/crie/5534847

**Published:** 2026-05-29

**Authors:** Tamer Draidi, Mohammad Khalil, Abed Kabaha, Rafat Zuriqi, Mohammad Masu’d

**Affiliations:** ^1^ Department of Medicine, Faculty of Medicine and Health Sciences, An-Najah National University, Nablus, State of Palestine, najah.edu

**Keywords:** ADAR, Aicardi–Goutières syndrome, central congenital hypothyroidism, infant cardiomyopathy, retinal detachment, transient hyperinsulinemic hypoglycemia, TSHB

## Abstract

**Background:**

Central congenital hypothyroidism (C‐CH) due to thyroid‐stimulating hormone beta (TSHB) variants is rare and often missed by thyroid‐stimulating hormone (TSH)–based neonatal screening. Adenosine deaminase acting on RNA (ADAR)‐related Aicardi–Goutières syndrome type 6 (AGS6) is an interferonopathy with early‐onset encephalopathy.

**Case Summary:**

A term small‐for‐gestational‐age male infant of consanguineous parents presented at 3 months with failure to thrive, generalized edema, transient hyperinsulinemic hypoglycemia and biventricular hypertrophy on echocardiography. Biochemistry showed profoundly low free thyroxine (FT4) and free triiodothyronine with inappropriately low TSH, consistent with central hypothyroidism, together with hypoalbuminemia and elevated liver enzymes. L‐thyroxine replacement and nutritional rehabilitation corrected hypoglycemia and were followed by complete normalization of cardiac structure and function on repeat echocardiography. Persistent global developmental delay, hypotonia and abnormal eye movements prompted neuro‐ophthalmological assessment, which demonstrated bilaterally small globes with peripheral retinal/choroidal detachment. Whole‐exome sequencing identified a homozygous pathogenic ADAR frameshift variant (NM_001111.5:c.2433_2434del) and a homozygous likely pathogenic TSHB variant (NM_000549.5:c.313T >C; p.Cys105Arg), confirming combined ADAR‐related encephalopathy and isolated C‐CH. At 8 months the child remained growth‐restricted with marked developmental delay but stable cardiorespiratory status.

**Conclusion:**

This case underscores the value of early genetic evaluation in infants with central hypothyroidism and syndromic features and of systematic cardiac surveillance in severe neonatal hypoglycemia to detect potentially reversible cardiomyopathy. To our knowledge, this is the first reported case of coexisting TSHB‐related C‐CH and ADAR‐related AGS6 in the same infant.

## 1. Introduction

Central congenital hypothyroidism (C‐CH) is a rare disorder in which insufficient thyrotropin stimulation of an otherwise normal thyroid gland leads to low circulating thyroxine, with low or inappropriately normal thyroid‐stimulating hormone (TSH) [1]. Because most newborn screening programs rely on elevated TSH, C‐CH is frequently missed and diagnosis is often delayed, increasing the risk of irreversible neurodevelopmental impairment [2].

Biallelic variants in the TSH beta (TSHB)‐subunit gene cause one form of isolated C‐CH, characterized biochemically by very low free thyroxine (FT4) with low or mildly elevated TSH and clinically by hypotonia, feeding difficulties and growth failure in early infancy [3]. Fewer than 40 affected individuals have been reported worldwide, and several distinct missense and truncating variants have been described, including p.Cys105Arg in the “seatbelt” region of the β‐subunit, a structurally important disulfide‐bonded loop that wraps around the common α‐subunit and is critical for heterodimer stability, receptor binding, and hormone bioactivity [3].

Aicardi–Goutières syndrome (AGS) is a monogenic type I interferonopathy presenting with early‐onset encephalopathy, intracranial calcifications, leukodystrophy and, variably, ocular abnormalities [4]. Pathogenic variants in adenosine deaminase acting on RNA (ADAR) define AGS type 6 (AGS6) and show striking phenotypic heterogeneity, from severe neonatal disease to milder later‐onset presentations [4].

We report an infant from consanguineous parents with homozygous variants in both TSHB and ADAR, presenting with central hypothyroidism, hyperinsulinemic hypoglycemia, transient biventricular hypertrophy and bilateral retinal/choroidal detachment. To our knowledge, this constellation has not previously been described. The case highlights the importance of early genetic testing and multidisciplinary cardiac, neurological and ophthalmological surveillance in infants who appear to have isolated C‐CH but exhibit syndromic features. We did not identify any previously published report describing this specific dual molecular diagnosis, and we therefore present this case as a novel association and discuss the mechanistic and diagnostic implications, particularly for settings that rely on TSH‐based newborn screening.

## 2. Case Presentation

### 2.1. Perinatal History

A male infant was born at 40 weeks’ gestation to healthy first‐cousin parents after an uncomplicated pregnancy. Fetal ultrasonography was reassuring. Delivery was by spontaneous vaginal birth; birth weight was 2.5 kg (small for gestational age), length was 48 cm and head circumference was 33 cm. Apgar scores were normal. He required admission to a neonatal unit for 14 days because of hyperbilirubinaemia treated with phototherapy, with no documented hypoglycemia or sepsis. After discharge he was exclusively breastfed. Routine newborn screening for congenital hypothyroidism in our setting relies on TSH measurement and therefore does not reliably detect C‐CH; accordingly, the newborn screening report was not available for review; however, since screening is TSH–based, it would not reliably detect C‐CH, and thyroid function testing was not performed during the admission for neonatal jaundice.

### 2.2. Presentation for 3 Months

Despite apparently adequate intake, weight gain remained poor, and the parents noted progressive puffiness of the face and lower limbs. At 3 months of age, he was admitted to a regional hospital with failure to thrive and generalized edema. On admission, he appeared pale and lethargic, with cool extremities and periorbital swelling. Weight was 2.5 kg, still close to birth weight; length was 50 cm. Cardiovascular examination revealed a regular pulse with a soft systolic murmur; there were no signs of respiratory distress. There was no history of hypoglycemic seizures, apnea, or documented convulsions prior to admission; symptoms were predominantly poor feeding, lethargy, and failure to thrive.

Capillary blood glucose was 30 mg/dL. A “critical sample” and full biochemical profile were obtained before intravenous dextrose. Laboratory tests showed plasma insulin of 8.9 µU/mL (published pediatric reference value, <49.7 µU/mL) [1], albumin 3.1 g/dL (published pediatric reference interval for age, 2.9–5.5 g/dL), AST 224 IU/L (published pediatric reference interval, 3–74 IU/L), ALT 76 IU/L (published pediatric reference interval, 5–50 IU/L), and creatine phosphokinase 449 IU/L (published pediatric reference interval for age, 0–440 IU/L) [5–8], with normal ammonia and lactate. These findings were consistent with transient hyperinsulinemic hypoglycemia with associated hepatic dysfunction and hypoalbuminemia. Ketone bodies and free fatty acids were not measured at the time of the critical sample; however, the detectable insulin level during profound hypoglycemia and the subsequent clinical course supported a diagnosis of transient hyperinsulinism. Blood gas analysis did not show metabolic acidosis (pH 7.47, pCO_2_ 28 mmHg, HCO_3_^−^ 22 mmol/L).

Thyroid function revealed profoundly low FT4 (0.09 ng/dL; published pediatric reference interval for age, 0.9–2.0 ng/dL) [9] and low FT3 (0.9 pg/mL) with inappropriately low TSH (0.6 mIU/L). Morning serum cortisol (20 µg/dL) was adequate for age [10]. The overall biochemical pattern supported central hypothyroidism rather than primary thyroid failure [1]. He commenced on intravenous dextrose and high‐energy enteral feeds. L‐thyroxine 25 µg once daily (~10 µg/kg/day) was initiated and gradually titrated. Hypoglycemic episodes resolved without the need for diazoxide or glucagon infusion, and edema improved with albumin correction and nutritional support. Resolution occurred in parallel with initiation of levothyroxine and nutritional rehabilitation, suggesting a combined endocrine–nutritional contribution to recovery.

### 2.3. Cardiac Assessment

Because of the presence of severe hypoglycemia and a systolic murmur, transthoracic echocardiography was performed in the neonatal unit. This demonstrated mild biventricular hypertrophy with preserved systolic function, normal chamber dimensions and no outflow‐tract obstruction or valvular disease. The findings were consistent with transient cardiomyopathy related to endocrine and metabolic disturbances [11].

At 6 months, shortly before scheduled brain magnetic resonance imaging (MRI) under anesthesia, repeat echocardiography at a tertiary cardiology center showed levocardia, atrial situs solitus, and concordant atrioventricular and ventriculo‐arterial connections. Ventricular dimensions and wall thickness were normal, with good systolic function. The only abnormality was a small patent foramen ovale with a trivial left‐to‐right shunt. The impression was “history of biventricular hypertrophy; currently normal cardiac structure and function,” and no restrictions were advised.

### 2.4. Endocrine Follow‐Up and Growth

By 5 months the infant remained markedly growth‐restricted: weight 4.1 kg and length 58 cm (both <3rd percentile). He was receiving a total weekly L‐thyroxine dose of 200 µg (25 µg on 4 days and 50 µg on 2 days). Clinical examinations showed frontal bossing, mild macrocephaly, axial hypotonia and head lag, but no coarse facies, goiter or peripheral edema.

Biochemical monitoring before discharge from the initial admission had shown biochemical over‐replacement (TSH 0.005 mIU/L with elevated FT4 2.86 ng/dL and FT3 7.14 pg/mL), leading to dose adjustment. The most recent available thyroid function tests were obtained at 4–5 months and demonstrated FT4 1.7 ng/dL and FT3 3.6 pg/mL with suppressed TSH, compatible with treated central hypothyroidism. No same‐day thyroid function panel was available from the exact 8‐month clinical review; however, based on the latest available biochemical assessment together with the absence of clinical features of hypo or hyperthyroidism, he was considered clinically euthyroid on therapy. Serial thyroid function results and treatment adjustments are summarized in Table 1.

**Table 1:** Serial thyroid function results and latest available endocrine follow‐up.

Age/time point	Treatment status	TSH (mIU/L)	FT4 (ng/dL)	FT3 (pg/mL)	Clinical/biochemical interpretation
3 months (presentation)	Before L‐thyroxine	0.6	0.09	0.9	Severe central hypothyroidism
Before discharge from initial admission	On L‐thyroxine, before dose adjustment	0.005	2.86	7.14	Biochemical over‐replacement
4–5 months follow‐up	After dose adjustment	Suppressed	1.7	3.6	Most recent available biochemical thyroid assessment; compatible with treated central hypothyroidism

### 2.5. Neurological and Ophthalmological Evaluation

Persistent abnormal eye movements, suspected squint and developmental delay (inability to roll or sit with support by 6 months) prompted referral to pediatric neurology and ophthalmology. At this time, he smiled and cooed but had poor visual fixation. There were no parental concerns regarding hearing, and auditory brainstem response (ABR) testing demonstrated normal hearing thresholds bilaterally.

Brain and orbit MRI with gadolinium at 6 months revealed relatively small ocular globes with bilateral peripheral folded membranes, more prominent on the left, compatible with peripheral choroidal or retinal detachment. There was benign enlargement of the subarachnoid space of infancy, a blooming artefact in both occipital horns of the lateral ventricles (more on the left) suggestive of remote intraventricular hemorrhage, but no hydrocephalus, intracranial mass, infarction or abnormal enhancement. Myelination was appropriate for age, and the intracranial arteries and dural venous sinuses were patent.

Ophthalmological examination confirmed bilateral optic‐disc swelling with truncated retinal vessels and suspected peripheral retinal detachment. The child tracked faces poorly and displayed abnormal tonic eye deviation. Examinations under anesthesia and electroretinography were planned but postponed because of logistical constraints.

At 8 months, a pediatric neurologist reviewed the infant after a 15‐min episode of repetitive left eyelid blinking associated with tachycardia, a concerning focal seizure. There was no fever or intercurrent illness. He could not roll or sit independently but was able to laugh, vocalize, and transfer objects between hands. Tone assessment revealed axial and peripheral hypotonia with preserved deep tendon reflexes. Awake electroencephalography showed no epileptiform discharges. The impression was a global developmental delay with axial hypotonia in the context of a probable underlying genetic/metabolic disorder; antiepileptic medication was deferred pending recurrence of seizures. Neurologic, ophthalmologic, audiologic, and neuroimaging findings are summarized in Table 2 for clarity.

**Table 2:** Summary of neurologic, ophthalmologic, audiologic, and neuroimaging findings.

Domain	Age/time point	Assessment	Main findings	Clinical impression
Visual behavior	~6 months	Clinical observation	Poor visual fixation; abnormal eye movements; suspected squint	Significant visual dysfunction
Hearing	10 September 2024	Auditory brainstem response (ABR)	Wave V obtained at 30 dBnHL bilaterally; normal hearing thresholds bilaterally	No objective hearing impairment detected
Neuroimaging	6 months	Brain and orbit MRI with gadolinium	Relatively small ocular globes; bilateral peripheral folded membranes, more prominent on the left, compatible with peripheral choroidal or retinal detachment; benign enlargement of the subarachnoid space of infancy; blooming artefact in both occipital horns suggestive of remote intraventricular hemorrhage; no hydrocephalus, intracranial mass, infarction, or abnormal enhancement; myelination appropriate for age	Major ocular structural abnormalities without hydrocephalus or active intracranial lesion
Ophthalmology	6 months	Specialist ophthalmologic examination	Bilateral optic‐disc swelling; truncated retinal vessels; suspected peripheral retinal detachment; poor face tracking; abnormal tonic eye deviation	Severe bilateral ophthalmologic involvement
Development/neurology	8 months	Pediatric neurology review	Unable to roll or sit independently; able to laugh, vocalize, and transfer objects between hands; axial and peripheral hypotonia with preserved deep tendon reflexes	Global developmental delay with hypotonia
Possible seizure event	8 months	Clinical event review	15‐min episode of repetitive left eyelid blinking associated with tachycardia	Concerning for focal seizure
EEG	8 months	Awake electroencephalography	No epileptiform discharges	Antiepileptic treatment deferred pending recurrence

### 2.6. Genetic Investigations

Given the combination of central hypothyroidism, transient hyperinsulinemic hypoglycemia, ocular malformations, developmental delay, and parental consanguinity, clinical trio‐based whole‐exome sequencing was undertaken at a regional genetics laboratory. Given the multisystem phenotype and parental consanguinity, we proceeded directly to trio whole‐exome sequencing rather than a targeted endocrine‐focused gene panel. Therefore, a predefined panel gene count was not applicable in this case. Variant interpretation was based on American College of Medical Genetics and Genomics criteria [12], and parental segregation analysis was used to support the molecular findings. Because the assay was performed as part of routine clinical care at an external laboratory, detailed run‐level technical parameters, including sequencing platform, read length, average sequencing depth, target‐region coverage statistics, and the full bioinformatics pipeline, were not available in the original laboratory report.

Analysis identified two homozygous variants. The first was an indel in ADAR (NM_001111.5: c.2433_2434del), predicted to introduce a premature stop codon in the ADAR 1 protein. Loss‐of‐function variants in ADAR are a recognized cause of AGS6, a type I interferonopathy [4]. The variant was classified as pathogenic according to American College of Medical Genetics and Genomics criteria.

The second variant was a homozygous missense change in TSHB (NM_000549.5: c.313T >C; p.Cys105Arg), located in the β‐subunit “seatbelt” region critical for proper folding and receptor binding. This variant has been reported in association with isolated C‐CH and was classified as likely pathogenic [13]. Segregation analysis showed that both parents were heterozygous carriers of the ADAR and TSHB variants, consistent with autosomal‐recessive inheritance of two distinct conditions. No clinically relevant copy‐number or structural variants were detected. Notably, both ADAR and TSHB are located on chromosome 1 at distinct loci, and parental consanguinity likely increased autozygosity and the probability of homozygous pathogenic variants.

### 2.7. Outcome and Follow‐Up

At the most recent review (8 months of age), the infant weighed 5.9 kg and measured 61 cm in length (both <3rd percentile); head circumference was 41.5 cm (just above −2 SD). He remained clinically euthyroid and normoglycaemic on L‐thyroxine monotherapy and specialized formula feeds, without diazoxide or other hypoglycemia treatments. No same‐day thyroid function tests were available at this visit; the latest available biochemical assessment had been obtained at 4–5 months and showed improved biochemical control on treatment. Cardiac examinations and repeat echocardiography were normal. However, he had persistent visual impairment and global developmental delay. He was referred for ongoing early‐intervention physiotherapy and occupational therapy, repeat detailed ophthalmic assessment, including examination under anesthesia, and multidisciplinary follow‐up by pediatric endocrinology, neurology, and clinical genetics.

## 3. Discussion

This case illustrates several important issues for clinicians caring for infants with central hypothyroidism.

### 3.1. Central Hypothyroidism due to TSHB

C‐CH is defined by low FT4 with low or “inappropriately normal” TSH, reflecting impaired hypothalamic–pituitary stimulation of an otherwise normal thyroid gland [1]. Because newborn screening programs typically use an elevated TSH cut‐off, infants with C‐CH may escape detection and present later with failure to thrive, hypothermia, or developmental delay [2].

Biallelic loss‐of‐function or missense variants in TSHB cause isolated C‐CH, usually with very low FT4 and absent or low‐normal TSH, while other pituitary hormone axes remain intact [3]. The p.Cys105Arg variant identified in our patient lies in a highly conserved cysteine residue of the “seatbelt” loop, which is essential for β‐subunit stability; experimental work suggests that substitutions at this position impair TSH secretion and bioactivity [13]. Our patient’s biochemical profile and robust clinical response to L‐thyroxine are consistent with this mechanism. Although thyroid dysfunction can occur in AGS, the biochemical pattern (low FT4 with inappropriately low TSH) together with the pathogenic TSHB variant supports a distinct, genetically determined isolated C‐CH in this infant [14].

### 3.2. ADAR‐Related Aicardi–Goutières‐Like Encephalopathy

The coexisting ADAR frameshift variant broadens the phenotype significantly. ADAR encodes a double‐stranded RNA–specific adenosine deaminase responsible for A‐to‐I RNA editing; pathogenic variants lead to accumulation of immunostimulatory RNA species and chronic activation of type I interferon signaling [4]. Clinically, ADAR‐related AGS (AGS6) presents with variable combinations of early‐onset encephalopathy, developmental delay, spasticity, seizures, intracranial calcifications and white‐matter abnormalities, often accompanied by chilblain‐like skin lesions or pigmentary changes [4]. Endocrine dysfunction has also been reported in AGS, including thyroid abnormalities and diabetes insipidus, supporting the need for ongoing endocrine surveillance in affected children [14].

Our patient did not show the classic neuroradiological hallmarks of AGS on early MRI: brain parenchyma and myelination was normal, and no calcifications or leukodystrophy were evident. Instead, the most striking features were global developmental delay, axial hypotonia, and severe ocular involvement with small globes and probable bilateral peripheral retinal or choroidal detachment. Ocular findings have been reported in some interferonopathies, including optic‐nerve and retinal changes, but are considered uncommon [15]. Our observation adds to the expanding spectrum of ADAR‐related disease and suggests that significant ophthalmologic pathology may precede or overshadow central nervous system abnormalities in some cases. Ocular involvement in AGS has been described (e.g., nystagmus, optic atrophy, cortical blindness, and congenital glaucoma), although comprehensive characterization remains limited [16].

### 3.3. Cardiovascular Manifestations of Endocrine and Metabolic Disease

Severe neonatal hypoglycemia due to hyperinsulinism is strongly associated with transient cardiac hypertrophy, typically involving the interventricular septum, which often normalizes as insulin levels fall and metabolic control improves [11]. Our patient presented profound hypoglycemia and inappropriately elevated insulin, consistent with transient hyperinsulinemic hypoglycemia, and had biventricular hypertrophy on initial echocardiography that fully resolved on follow‐up once euglycaemia and euthyroidism were achieved [17, 18]. Although insulin‐mediated hypertrophy classically predominates in the interventricular septum, diffuse/biventricular hypertrophy has also been described in hyperinsulinism, and the absence of outflow obstruction together with rapid reversibility in our patient supports a predominantly metabolic/hormonal mechanism rather than primary sarcomeric hypertrophic cardiomyopathy.

Although C‐CH is classically associated with fasting intolerance and hypoglycemia, our patient demonstrated inappropriately elevated insulin during hypoglycemia, supporting a diagnosis of transient hyperinsulinemic hypoglycemia. We speculate that impaired counter‐regulatory responses in hypothyroidism, together with hepatic dysfunction and limited nutritional reserves, may have lowered the threshold for insulin‐mediated hypoglycemia. Resolution occurred with nutritional rehabilitation and initiation of L‐thyroxine, without the need for diazoxide, suggesting a transient functional hyperinsulinism rather than a persistent monogenic hyperinsulinism syndrome.

Congenital hypothyroidism itself can impair myocardial function and has been associated with cardiac hypertrophy and pericardial effusion in infants [19]. The overlapping contributions of hyperinsulinism and hypothyroidism likely explain the transient cardiomyopathy observed in this case. For cardiologists, this underscores the need for cardiac imaging in infants with severe endocrine disturbances and for systematic metabolic and endocrine evaluation in infants presenting with otherwise unexplained hypertrophic cardiomyopathy [20, 21].

### 3.4. Diagnostic Yield of Early Exome Sequencing

The coexistence of two ultrarare autosomal‐recessive disorders in the same child illustrates the utility of early next‐generation sequencing in multisystem disease, particularly in populations with high consanguinity. Traditional sequential testing might have targeted only pituitary or thyroid genes in the context of central hypothyroidism, potentially missing the ADAR variant and leading clinicians to attribute neurological and ophthalmological abnormalities solely to delayed thyroid treatment [13]. Identification of both pathogenic variants enabled precise molecular diagnosis, informed prognosis and facilitated accurate recurrence‐risk counseling for the family.

In similar presentations, a tiered genetic strategy may be considered. In infants with biochemically confirmed isolated C‐CH and no additional syndromic features, a targeted pituitary/endocrine gene panel (including TSHB and other known causes of isolated or combined pituitary hormone deficiency) may be an efficient first step. However, in consanguineous families or when central hypothyroidism coexists with hypoglycemia, developmental delay, ocular anomalies or other organ involvement, first‐line trio whole‐exome sequencing provides a higher diagnostic yield and may shorten time to diagnosis [22].

### 3.5. Multidisciplinary Management

Finally, this case underscores the importance of coordinated multidisciplinary care. Close collaboration between pediatric endocrinology, cardiology, neurology, ophthalmology and clinical genetics allowed timely optimization of thyroid replacement, cardiac surveillance, neurodevelopmental assessment, and genetic work‐up despite limited resources. Such integrated care pathways are essential to improve outcomes for infants with complex genetic endocrine–cardio–neurological syndromes.

## 4. Limitations

As a single‐patient report, causal attribution of individual manifestations (e.g., hypoglycemia and myocardial hypertrophy) to one endocrine or genetic mechanism is limited, and some metabolic markers (e.g., ketone bodies/free fatty acids) were not obtained during hypoglycemic episodes. In addition, reference intervals vary across laboratories and assays. Because the exome analysis was performed as an external clinical diagnostic test, full assay‐level sequencing metrics and the detailed bioinformatics workflow were not available from the original laboratory report. These limitations should be considered when generalizing the observations from this case.

## 5. Conclusion

We describe an infant with C‐CH due to homozygous TSHB p.Cys105Arg and ADAR‐related Aicardi–Goutières‐like encephalopathy, who presented with failure to thrive, hyperinsulinemic hypoglycemia, transient biventricular hypertrophy, and bilateral retinal/choroidal detachment. The case highlights several key lessons, as follows:

1. Central hypothyroidism should be suspected when severe hypothyroxinaemia coexists with low or normal TSH, especially in infants with growth failure or hypoglycemia.
2. Severe neonatal hypoglycemia warrants cardiac evaluation, as endocrine‐mediated cardiomyopathy may be reversible with appropriate metabolic and hormonal treatment.
3. Early exome sequencing is invaluable in infants with central hypothyroidism and syndromic features, particularly in consanguineous families, and can reveal dual genetic diagnoses that shape prognosis and counseling.
4. Multidisciplinary, longitudinal follow‐up involving endocrinology, cardiology, neurology, ophthalmology, and genetics is crucial to optimize outcomes in such complex cases.

Interpretation of biochemical results should take into account population‐ and age‐appropriate reference intervals whenever available, and longitudinal follow‐up is best performed using the same laboratory platform or laboratory whenever feasible to improve consistency and comparability of serial measurements.

## Acknowledgments

No artificial intelligence or AI‐assisted technologies were used in the drafting, analysis, interpretation, or preparation of this manuscript.

## Funding

This work received no funding from governmental, commercial, or nonprofit organizations.

## Consent

Written informed consent for publication of the clinical details and anonymized images was obtained from the child’s legal guardians in accordance with institutional policies and the Declaration of Helsinki.

## Conflicts of Interest

The authors declare no conflicts of interest.

## Data Availability

The data that support the findings of this study are available upon request from the corresponding author. The data are not publicly available due to privacy or ethical restrictions.
